# Phospholipid Scramblase 1, an interferon-regulated gene located at 3q23, is regulated by SnoN/SkiL in ovarian cancer cells

**DOI:** 10.1186/1476-4598-12-32

**Published:** 2013-04-26

**Authors:** Karthik M Kodigepalli, Pavana Anur, Paul Spellman, Peter J Sims, Meera Nanjundan

**Affiliations:** 1 Department of Cell Biology, Microbiology, and Molecular Biology, University of South Florida, 4202 East Fowler Avenue, ISA2015, Tampa, FL 33620, USA; 2 Department of Molecular and Medical Genetics, Oregon Health and Science University, 3181 SW Sam Jackson Park Road, Portland, OR, USA; 3 University of Rochester Medical Center, School of Medicine and Dentistry, Rochester, NY, USA

**Keywords:** SnoN/SkiL, Phospholipid scramblase, PLSCR1, Arsenic trioxide, Interferon, TGF-β, 3q26.2, 3q23, Ovarian cancer

## Abstract

**Background:**

Treatment of advanced stage ovarian cancer continues to be challenging due to acquired drug resistance and lack of early stage biomarkers. Genes identified to be aberrantly expressed at the 3q26.2 locus (i.e. SnoN/SkiL) have been implicated in ovarian cancer pathophysiology. We have previously shown that SnoN expression is increased in advanced stage ovarian cancers and alters cellular response to arsenic trioxide (As_2_O_3_).

**Findings:**

We now demonstrate increased DNA copy number levels (TCGA data) of phospholipid scramblase 1 (PLSCR1, located at 3q23) whose transcript expression in ovarian cell lines is highly correlated with SnoN mRNA. Interestingly, SnoN can modulate PLSCR1 mRNA levels in the absence/presence of interferon (IFN-2α). Both IFN-2α and As_2_O_3_ treatment can modulate PLSCR1 mRNA levels in ovarian carcinoma cells. However, SnoN siRNA does not lead to altered PLSCR1 protein implicating other events needed to modulate its protein levels. In addition, we report that PLSCR1 can modulate aspects of the As_2_O_3_ cellular response.

**Conclusions:**

Our findings warrant further investigation into the role of PLSCR1 in ovarian cancer development and chemoresistance.

## Findings

Epithelial ovarian cancer represents the most common gynecological cancer in women with an unfortunate high mortality rate due to acquired chemotherapeutic resistance [1]. Our earlier published studies indicate that the 3q26.2 chromosomal region is highly amplified in ovarian cancers [2] and harbors various oncogenes including EVI1 [2], PKCι [3], and SnoN/SkiL [4]. In particular, we previously demonstrated that SnoN, a negative transcriptional regulator of TGFβ signaling, modulates the pro-survival autophagic pathway in response to arsenic trioxide (As_2_O_3_), a chemotherapeutic agent used in the treatment of acute promyelocytic leukemia (APL) [5]. Interestingly, there are reports which indicate that genes located at and proximal to the 3q26 locus may regulate each other. For instance, both EVI1 and PIK3CA can regulate SnoN expression [6,7]. Herein, we now report that the expression of phospholipid scramblase 1 (PLSCR1), located at 3q23, can be modulated via SnoN. PLSCR1 has been implicated in maintaining plasma membrane lipid asymmetry, regulating growth factor signaling pathways, in modulating tumor growth in mouse xenograft models [8], and cancer development [9,10]. The role of PLSCR1 in ovarian cancer and in modulating response to chemotherapeutic agents has yet to be fully understood.

**Figure 1:**
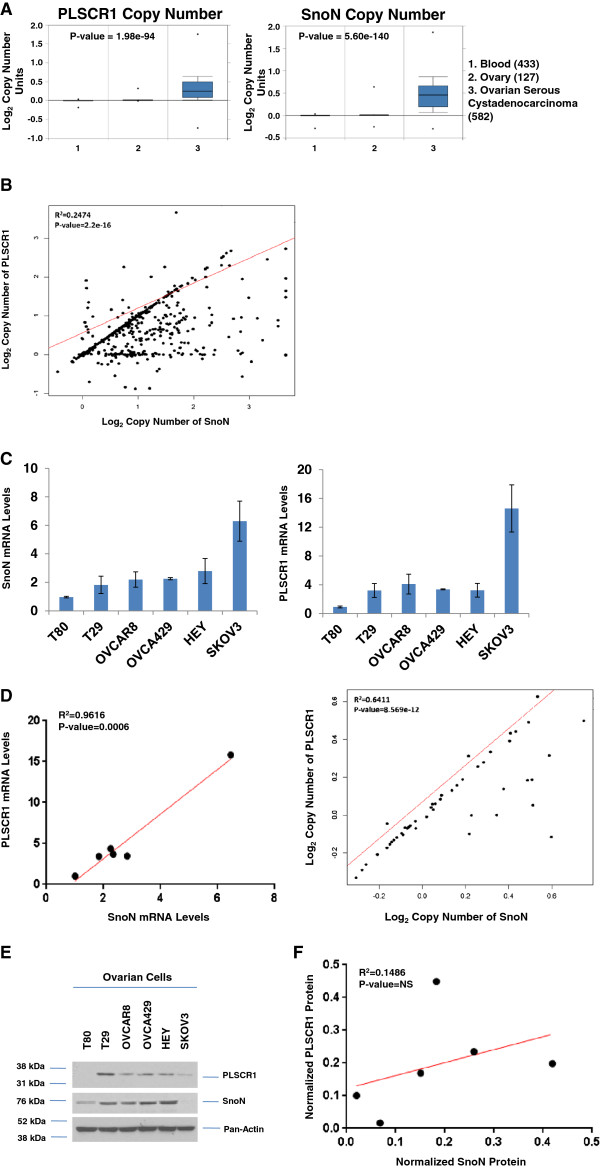
**Concordance between SnoN and PLSCR1 RNA expression in ovarian cancer cell lines. **(**A**) Oncomine analysis of the Ovarian TCGA data set shows increased copy number of PLSCR1 (left panel) and SnoN (right panel) in ovarian serous cystadenocarcinomas (582 specimens) in contrast to normal ovary (127 specimens). (**B**) Correlation analysis between PLSCR1 and SnoN copy number in ovarian cancer specimens. (**C**) RNA was harvested from normal (T80 and T29) and malignant (OVCAR8, OVCA429, HEY, and SKOV3) ovarian cell lines. PLSCR1 mRNA levels were quantified by quantitative PCR. The results are displayed as relative RNA-fold change. Results are representative of duplicate experiments. (**D**) Correlation analyses between PLSCR1 and SnoN RNA and DNA copy number in ovarian cancer cell lines. (**E**) Western blotting was performed using antibodies against the proteins indicated using cell lysates isolated from the cell lines described in (**C**). Results are representative of duplicate experiments. (**F**) Correlation analyses between PLSCR1 and SnoN protein expression.

Our previous aCGH studies from 235 ovarian cancer patient samples demonstrated that SnoN was increased at the DNA copy number level [4]. We now identify through Oncomine bioinformatic analyses (ovarian TCGA dataset (https://tcga-data.nci.nih.gov.tcga/) that the DNA copy number levels of PLSCR1 in addition to SnoN are altered similarly (Figure 1A). Furthermore, using cBioportal [11], we identified that SnoN is amplified in 31% of the cases whereas PLSCR1 is amplified in 13% of the cases (70 out of 570 samples amplified both genes). To determine whether SnoN and PLSCR1 genes are co-amplified, we performed linear regression on copy number variation (CNV) estimates (Additional file 1: Methods and Materials) for SnoN and PLSCR1 genes in R (http://www.R-project.org/) (Figure 1B). In ovarian cancers with PLSCR1 amplification, SnoN is gained. When SnoN is amplified, PLSCR1 is only gained in ~33% of the samples (R^2^ = 0.2474) (Figure 1B). We next evaluated the RNA and protein levels of PLSCR1 in various normal and malignant ovarian cell lines via real-time PCR and western analysis. Similar to SnoN, PLSCR1 expression was low in normal immortalized T80 ovarian cells and highly expressed in the ovarian cancer cell lines (Figure 1C and E). Although PLSCR1 and SnoN expression were highly correlated (via linear regression) at the RNA level (Figure 1D), there was a discordance at the protein level (Figure 1F) which has been reported previously for other genes [12,13]. Furthermore, the DNA copy number of PLSCR1 and SnoN is nearly always the same in ovarian cancer cell lines (R^2^ = 0.6411) (Additional file 2: Table S1). Collectively, these results demonstrate that, PLSCR1 is increased at the DNA and RNA levels in ovarian cancers and cell lines in comparison to normal cells, similar to SnoN, and can be co-amplified in a certain proportion of ovarian cancer specimens. However, there likely exist additional levels of regulation which contribute to modulating PLSCR1 protein levels.

**Figure 2:**
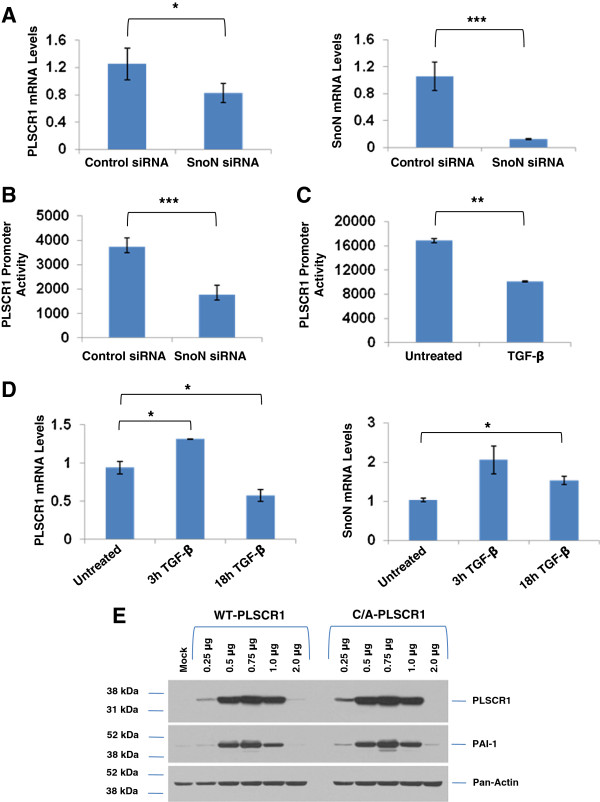
**Knockdown of SnoN Reduces PLSCR1 mRNA Levels. **(**A**) RNA was isolated from HEY cells 48-hours post-transfection with SnoN siRNA. PLSCR1 (left panel) and SnoN (right panel) mRNA levels were quantified by real-time PCR. Results are representative of duplicate experiments. (**B**) T80 cells were transfected with the PLSCR1 promoter followed by either SnoN knockdown (**B**) or 24 h TGFβ treatment (**C**). Cell lysates were harvested and luciferase activity was quantified. Results are representative of duplicate experiments. (**D**) RNA was isolated from T80 cells treated with TGFβ (3 and 18 h). PLSCR1 (left panel) and SnoN (right panel) mRNA levels were quantified by real-time PCR. Results are representative of duplicate experiments. (**E**) T80 cells were mock transfected or transiently transfected with either wild type PLSCR1 (WT-PLSCR1, 0.25 μg to 2.0 μg) or C/A PLSCR1 mutant (C/A-PLSCR1, 0.25 μg to 2.0 μg). Cell lysates were analyzed by western blotting for the proteins indicated. Results are representative of duplicate experiments.

Since PLSCR1 is located in close proximity to SnoN at the 3q locus [2], we next assessed whether SnoN could modulate PLSCR1 expression. To address this question, we reduced SnoN expression via siRNA in HEY ovarian carcinoma cells (cell line used previously to investigate role of SnoN [5] and PLSCR1 [8]); this was followed by quantitation of PLSCR1 mRNA levels via real-time PCR. Upon SnoN knockdown (~88% and 95% at RNA and protein level, respectively), we observed a significant reduction (~35%) in PLSCR1 mRNA (Figure 2A) implicating SnoN in the regulation of PLSCR1 transcription. These results were validated by utilizing the PLSCR1 promoter upon SnoN knockdown in T80 cells (Figure 2B) or with TGFβ (50 pM) (Figure 2C); both conditions led to a marked reduction in PLSCR1 promoter activity suggesting that activation of the TGFβ signaling cascade downregulates PLSCR1 expression. PLSCR1 mRNA levels were also down-regulated following 24 h TGFβ treatment (Figure 2D, left panel). SnoN mRNA has been previously shown to increase 1–3 hours post-TGFβ treatment [4] (Figure 2D, right panel) implicating discordance between SnoN and PLSCR1 mRNA levels with TGFβ. Intriguingly, overexpression of both the wild type and C/A PLSCR1 mutant (which localizes to the nuclear compartment [14]) in T80 cells led to a marked induction of plasminogen activator inhibitor-1 (PAI-1) expression (Figure 2E); these results suggest that PLSCR1 could modulate TGFβ cellular responses, similar to SnoN [4].

**Figure 3:**
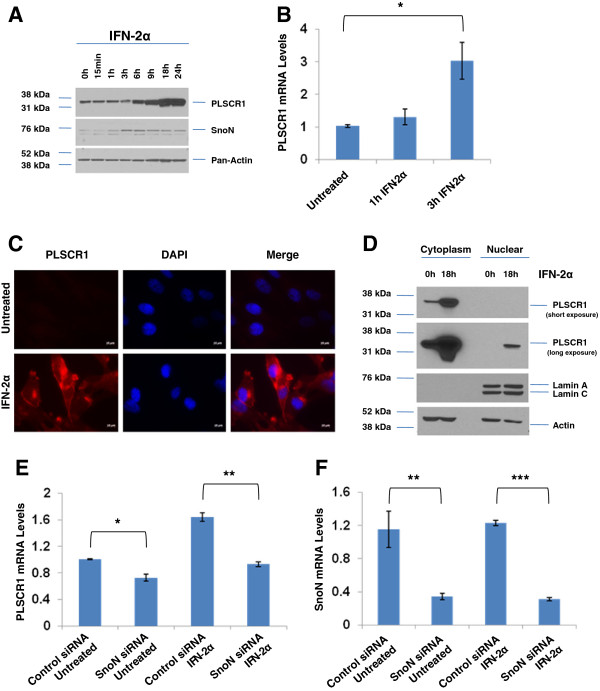
**SnoN modulates IFN-2α-mediated induction of PLSCR1 mRNA. **(**A**) HEY cells were treated with 3000 IU/ml IFN-2α (15 min – 24 h). Cell lysates were harvested followed by western blotting for the indicated proteins. Results are representative of triplicate experiments. (**B**) HEY cells were treated with 3000 IU/ml IFN-2α (0, 1, and 3 hours). RNA was isolated followed by real-time PCR analysis to quantify PLSCR1 mRNA levels. Results are representative of duplicate experiments. HEY cells were treated with 3000 IU/ml IFN-2α (18 h) followed by immunofluorescence staining (**C**) or subcellular fractionation of cytoplasmic and nuclear fractions (**D**) which were analyzed by western blotting for the proteins indicated. Results are representative of duplicate experiments. (**E**) HEY cells were transfected with non-targeting or SnoN siRNA followed by IFN-2α treatment (3 h). Forty-eight hours post-transfection, total RNA was isolated followed by quantitation of PLSCR1 (**E**) and SnoN (**F**) mRNA by real-time PCR. Results are representative of duplicate experiments.

Since PLSCR1 is an interferon (IFN)-inducible gene [15,16], we next determined whether SnoN could, in part, modulate PLSCR1 expression upon IFN-2α treatment. Supporting previous reports, treatment of HEY cells with IFN-2α (3000 IU/ml) led to a dramatic increase in PLSCR1 protein from 6 up to 24 hours (Figure 3A, 3.7-fold) and RNA (Figure 3B, 2.9-fold). Similar results were also observed in a series of IFN-resistant and sensitive pancreatic cancer cell lines (Additional file 3: Figure S1, A-F). Strikingly, SnoN protein was induced (in the absence of SnoN mRNA changes (Additional file 4: Figure S2, A)) at 3 h post-IFN treatment; changes in SnoN occurred prior to those observed in PLSCR1. These results implicate SnoN in the transcriptional regulation of PLSCR1 expression upon IFN treatment. In addition, we noted that the induced PLSCR1 localized predominantly at the plasma membrane in HEY cells (Figure 3C and D), assessed via immunofluorescence and subcellular fractionation (with a very minor fraction localizing to the nuclear compartment). Since chemotherapeutic agents can generate intracellular reactive oxygen species (ROS) which modulates expression levels of various proteins [5], we next assessed whether the changes we observed in PLSCR1 and SnoN expression with IFN were due to ROS. Thus, we co-treated HEY cells with N-acetyl-L-cysteine (NAC), an anti-oxidant free radical scavenger, together with IFN for 9 h. However, there were no marked changes in PLSCR1 and SnoN protein levels in the presence of NAC (Additional file 4: Figure S2, B); these results suggest that the IFN-induced changes in SnoN and PLSCR1 may be independent of ROS. Strikingly, knockdown of SnoN levels (via siRNA) in HEY cells in the presence of IFN-2α (6 h, 3000 IU/ml) not only effectively reduced SnoN levels but also PLSCR1 RNA (Figure 3E and F, 1.8-fold). However, changes in PLSCR1 protein were again not detected with IFN-treatment following SnoN siRNA (Additional file 4: Figure S2, C); we propose that this could be due to the long half-life of PLSCR1 protein (assessed utilizing cycloheximide (CHX), an inhibitor of protein translation (results not shown)) or additional mechanisms needed to contribute with SnoN to modulate PLSCR1 protein.

**Figure 4:**
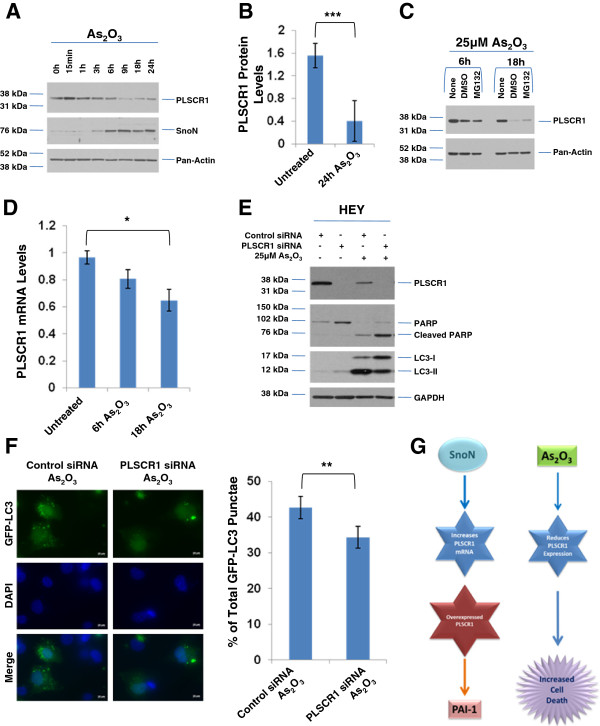
**PLSCR1 modulates As**_**2**_**O**_**3**_**-induced cell death response in HEY ovarian carcinoma cells. **(**A**) HEY cells were treated with 25 μM As_2_O_3 _(15 min - 24 h) followed by harvesting of cell lysates and analyses via western blotting for the indicated proteins. Results are representative of triplicate experiments. (**B**) Densitometric analysis of PLSCR1 protein levels of data presented in (**A**). (**C**) HEY cells were treated with 25 μM As_2_O_3 _(6 or 18 h) in the absence or presence of MG132 (5 μM) followed by harvesting of cell lysates and analyses via western blotting for the indicated proteins. Results are representative of duplicate experiments. (**D**) HEY cells were treated with 25 μM As_2_O_3 _(6 or 18 h). RNA was isolated and analyzed for PLSCR1 mRNA levels by real-time PCR. Results are representative of duplicate experiments. (**E**) Cell lysates were collected from HEY cells treated with non-targeting or PLSCR1 siRNA in the absence or presence of 25 μM As_2_O_3 _(18 h). Western blotting was performed for the proteins indicated. Results are representative of triplicate experiments. (**F**) Cells were transfected with pEGFP-LC3. Twenty-four hours post transfection, cells were treated with non-targeting and PLSCR1 siRNA followed by treatment with 10 μM As_2_O_3_. Immunofluorescence was performed (left panel) and the data was quantitated (bar graphs, right panel) by counting the number of cells positive for EGFP-LC3 punctae. Results are representative of duplicate experiments. (**G**) A schematic showing regulation of PLSCR1 mRNA by SnoN and As_2_O_3 _(and its effect on the cell death response) in HEY cells. In addition to the downstream effect of overexpressed PLSCR1 on PAI-1, a TGFβ target gene, in T80 cells.

We have previously demonstrated the role of As_2_O_3_ as an effective chemotherapeutic agent inducing cell death in ovarian cancer cells, antagonized by autophagy mediated by SnoN induction [5]. We first assessed whether PLSCR1 protein is altered upon As_2_O_3_ treatment in ovarian cancer cells. In this regard, we treated HEY cells with 25 μM As_2_O_3_ (0 – 24 h). In contrast to SnoN (increasing between 6 – 24 h), we noted a marked reduction in PLSCR1 protein (~75% reduction) (Figure 4A and B). We next determined whether this reduction in PLSCR1 protein was due to proteasomal degradation via the use of MG132 (proteasome inhibitor). Co-treatment of HEY cells with 5 and 25 μM As_2_O_3_ for 6 h and 18 h with 5 μM MG132 did not lead to a significant recovery in PLSCR1 levels; these results suggest a mechanism of PLSCR1 protein regulation independent of the proteasome (Figure 4C). Indeed, As_2_O_3_ also alters PLSCR1 RNA levels which might together reflect As_2_O_3_-induced transcriptional regulation of PLSCR1 (Figure 4D). In order to determine whether PLSCR1 plays a role in modulating As_2_O_3_–induced cell death response in ovarian cancer cells, we reduced PLSCR1 expression via siRNA. Upon knockdown of PLSCR1 in the presence of As_2_O_3_, we observed a marked increase in the levels of caspase-3 activity (results not shown) as well as cleaved PARP ((7.9-fold) a marker of apoptosis, Figure 4E) concurrent with reduction in LC3-II ((2.5-fold), a marker of autophagy, Figure 4E) validated by GFP-LC3 autophagy assays ((~20%), Figure 4F). Similar to SnoN [5], these results suggest that PLSCR1 may contribute to the As_2_O_3_-induced apoptotic and autophagic response.

In the current study, we demonstrate that PLSCR1 and SnoN DNA copy number as well as their RNA levels are correlated. By modulating SnoN expression, PLSCR1 mRNA levels appear to be co-regulated (Figure 4G). Of interest, SnoN knockdown does not alter PLSCR1 protein possibly suggesting that other mediators are involved in its regulation. Nonetheless, similar to SnoN, reduction in PLSCR1 levels appears to increase the cellular sensitivity to As_2_O_3_. Whether PLSCR1 modulates sensitivity to carboplatin/paclitaxel or whether the effects of As_2_O_3_ and TGFβ are mediated via IFN remain to be investigated. Thus, further investigations are warranted to delve into the significance of these findings in ovarian cancer development and chemoresistance.

## Abbreviations

PLSCR1: Phospholipid Scramblase 1; SnoN/SkiL: Ski Related Novel Protein N; EVI1: Ecotropic Viral Integration Site-1; PIK3CA: phosphatidylinositol-3-kinase catalytic subunit-α; PKCι: Protein Kinase C iota; aCGH: Array Comparative Genomic Hybridization; TCGA: The Cancer Genome Atlas; PAI-1: Plasminogen Activator Inhibitor-1; TGFβ: Transforming Growth Factor-β; As2O3: Arsenic trioxide; CHX: Cycloheximide; IFN: Interferon; ROS: Reactive Oxygen Species; NAC: N-Acetyl-L-Cysteine; LC3: Microtubule-associated protein light chain 3; GFP: Green fluorescent protein; PI: Propidium Iodide; PARP: Poly-ADP Ribose Polymerase; T80: Immortalized (LTAg/hTERT) normal ovarian surface epithelial cells; APL: Acute Promyelocytic Leukemia.

## Competing interests

The authors declare that they have no competing interests.

## Authors’ contributions

MN conceived and supervised the study. PJS developed the following constructs for use in this work: pGL3-PLSCR1 promoter, wild type PLSCR1 in pcDNA3.1, and C/A PLSCR1 mutant in pcDNA3.1. KMK, PA, PS, and MN performed the research and analyzed the data. KMK and MN co-wrote the paper and all authors approved the final manuscript.

## Supplementary Material

Additional file 1 Materials and Methods. Click here for file

Additional file 2: Table S1 DNA Copy Number Variation in PLSCR1 and SnoN across multiple ovarian cancer cell lines. Forty-seven ovarian cancer cell lines were assessed for CNV in PLSCR1 and SnoN. There does not appear to be significant copy number changes for both genes in the cell lines presented where the copy number is nearly invariable from the normal copy number. Click here for file

Additional file 3: Figure S1 Induction of PLSCR1 protein expression in response to IFN-2α in a series of resistant and sensitive pancreatic cancer cell lines. (A) IFN-2α treatment of AsPC-1 cells and PANC-1 cells. (B) IFN-2α treatment of BxPC-3 cells and MIA PaCa-2 cells. Cell lysates were harvested from cell lines described in (A) and (B) followed by western analyses for the indicated antibodies. (C) Cell lysates were harvested from IFN-2α treated MIA PaCa-2 cells followed by western analyses for the indicated antibodies. (D) RNA was isolated from IFN-2α treated MIA PaCa-2 cells followed by real-time PCR analyses to quantify PLSCR1 mRNA levels. (E) and (F) Growth assays were performed in AsPC-1, PANC-1, BxPC-3, and MIA PaCa-2 cells in response to IFN-2α treatment. Click here for file

Additional file 4: Figure S2 No effect on PLSCR1 protein levels upon NAC treatment or SnoN knockdown in the absence/presence of IFN-2α. (A) HEY cells were treated with 3000 IU/ml IFN-2α (0, 1, 3 hours). RNA was isolated followed by real-time PCR analysis to quantify SnoN mRNA levels. (B) HEY cells were treated with IFN-2α, NAC, or IFN-2α in combination with NAC at the specified doses. Cell lysates were harvested and analyzed by western blotting analyses for the indicated antibodies. (C) HEY cells were transfected with SnoN siRNA and treated with/without IFN-2α. Cell lysates were harvested and western analyses performed for the indicated antibodies. Click here for file
