# Supplementary material for: Phospholipid Scramblase 1, an interferon-regulated gene located at 3q23, is regulated by SnoN/SkiL in ovarian cancer cells
Source: Mol Cancer. 2013 Apr 26;12:32. doi: 10.1186/1476-4598-12-32 (PMC3644492; doi:10.1186/1476-4598-12-32)
Supplement: Additional file 1 — Materials and Methods. [file 1476-4598-12-32-S1.docx]

**Additional File 1** Materials and Methods

*Cell culture and treatments*

Immortalized (LTAg/hTERT) normal ovarian surface epithelial cells (T80), HEY, BxPC-3, and AsPC-1 cells were cultured in RPMI 1640 media supplemented with 8% FBS and penicillin/streptomycin. PANC-1 and MIA PaCa-2 cells were cultured in DMEM media supplemented with 8% FBS and penicillin/streptomycin.

Cells were seeded at 250,000 cells in each well of a 6-well plate. Following 24 hours allowing cell adherence, cells were then treated with 50pM TGF-β (Calbiochem, CA, USA), 3000 – 10,000IU/ml IFN-2α (MBL International, MA, USA), 10 or 25μM As_2_O_3_ (Sigma-Aldrich, MO, USA), or 5μM MG132 (Alexis Biochemicals, NY, USA) for the appropriate times. Dimethyl sulfoxide (DMSO) was included as appropriate in control or untreated conditions at a final concentration of 0.05% (i.e. MG132).

*siRNA mediated gene knockdown*

Cells were seeded at 250,000 cells per well of a 6-well plate. Following adherence, cells were transfected with non-targeting control siRNA (D-001810-10, Dharmacon, CO, USA) or siRNA targeting PLSCR1 (L-003729-00) using Dharmafect I (Dharmacon) as previously described [[1](#_ENREF_1)].

*Overexpression of wild type and C/A mutant PLSCR1*

pcDNA3.1 vectors containing the coding sequence of wild type (WT) and C/A mutant PLSCR1 as well as empty pcDNA3.1 vector were transiently transfected using Fugene HD (Roche, WI, USA) into T80 cells as previously described [[2](#_ENREF_2)].

*Protein isolation, SDS-PAGE, and western analysis*

Following cell lysis (1% Triton X-100, 50mM HEPES, 150mM NaCl, 1mM MgCl_2,_ 1mM EGTA, 10% glycerol, and protease inhibitor cocktail (Roche, WI, USA)) for 1 h at 4^o^C, lysates were harvested, and centrifuged at 14,000 rpm for 10 min at 4^o^C. Appropriate SDS-PAGE gels were utilized (10%) for protein separation followed by transfer onto PVDF membranes. Western blotting was performed according to previous publications [[1](#_ENREF_1)]. PLSCR1 mouse monoclonal (1:500 dilution, sc-59645) and SnoN rabbit polyclonal (1:1000 dilution, sc-9141) were obtained from Santa Cruz Biotechnology (CA, USA). PAI-1 mouse monoclonal (1:500 dilution, #612024) was obtained from BD Biosciences (CA, USA). p21 mouse monoclonal (1:250 dilution, #2946), PARP rabbit polyclonal (1:1000 dilution, #9542), LC3 rabbit polyclonal (1:1000 dilution, #2775), Lamin A/C rabbit polyclonal (1:1000 dilution, #4777), and pan-Actin rabbit polyclonal (1:1000 dilution, #4968) antibodies were obtained from Cell Signaling Technology (MA, USA).

*Quantitative PCR*

RNA was isolated using the RNeasy kit (Qiagen, CA, USA) and quantified using Nanodrop. Quantitative PCR was performed using the One-Step PCR master mix (Applied Biosystems, CA, USA), primers/probes (Assays-by-Design) for detection of PLSCR1 (Hs01062171_m1) and SnoN (Hs01045418_m1), and the One-Step-Plus Applied Biosystems Detection System. β-actin was used as the endogenous reference control. The PCR conditions and correlative method of analyses were as previously described [[1](#_ENREF_1)].

*Luciferase promoter assays*

The luciferase vector (pGL3) containing the PLSCR1 promoter was transfected into T80 cells using Fugene HD. This was followed by either SnoN siRNA or TGF-β treatment (as described above). The promoter activity of PLSCR1 was determined by measuring luciferase activity (Promega, WI, USA) and a BioTek plate reader as previously described [[3](#_ENREF_3)].

*Indirect immunofluorescence*

Cells were seeded onto glass coverslips, allowed to adhere, and treated with IFN-2α for 18 h. Cells were then fixed with 4% formaldehyde (in PBS) for 30 min, washed In PBS, then blocked for 1 h (PBS containing 5% goat serum, and 0.1% Triton X-100), and incubated overnight at 4°C with anti-PLSCR1 mouse monoclonal antibody (1:50 dilution (sc-59645, 1E9 mouse monoclonal) in PBS containing 1% goat serum and 0.1% Triton X-100). Cells were washed and then incubated with Cy3-conjugated goat-anti-mouse antibody (1:500 dilution, Invitrogen, CA, USA) for 1 h at room temperature. Following PBS washes, coverslips were mounted onto glass slides with anti-fade DAPI nuclear stain.

For assessing EGFP-LC3 localization, cells were transfected with pEGFP-LC3 (Addgene, MA, USA) followed by PLSCR1 siRNA transfection and cellular treatment with 10μM As_2_O_3_ (18 h). Cells were fixed and blocked prior to mounting onto glass slides with anti-fade DAPI nuclear stain. All slides were analyzed using an inverted Zeiss fluorescence microscope (Moffitt Cancer Center, Microscopy Core, Tampa, Florida) as previously described [[1](#_ENREF_1)].

*Determination of copy number variation (CNV) estimates in ovarian cancers and ovarian cancer cell lines*

To determine if SnoN and PLSCR1 are co-amplified we used Copy Number Variation (CNV) estimates for these two genes from 559 patients in TCGA. GISTIC2 [[4](#_ENREF_4)] pipeline was used to generate CNV estimates. In addition we also looked at CNV estimates from 47 ovarian cancer cell lines. Circular Binary Segmentation (CBS) algorithm was performed on microarray data to obtain segmented CNV estimates [[5](#_ENREF_5)]. These were further mapped to genes to obtain-gene level estimates. As there is no matched normal for the cell lines, average copy number data from a set of ~20 HapMap samples was utilized to represent normal for estimation of CNV in ovarian cancer cell lines. To determine co-amplification of SnoN and PLSCR1 genes, we performed linear regression on CNV estimates for SnoN and PLSCR1 genes in R (http://www.R-project.org).

*Statistical analyses*

Experiments were performed at least in duplicate (n = 2) unless otherwise indicated (please refer to Figure Legends). Error bars represent standard deviations (on bar graphs). P-values were determined according to student’s T-test (* refers to p<0.05; ** refers to p<0.01; *** refers to p<0.001). For results presented in Figure 1D and 1F, R^2^ values (and associated p-values) were determined by Pearsons’ correlation using Prism 6 (GraphPad). Western blots were analyzed by densitometric analyses using Image J program (Image Processing and Analysis in Java, NIH Image Software, http://rsb/info/nioh/gov.ij/).

**Additional File 1 References**

**1.** Smith DM, Patel S, Raffoul F, Haller E, Mills GB, Nanjundan M: **Arsenic trioxide induces a beclin-1-independent autophagic pathway via modulation of SnoN/SkiL expression in ovarian carcinoma cells.** *Cell death and differentiation* 2010, **17**(12):1867-1881.

**2.** Nanjundan M, Nakayama Y, Cheng KW, Lahad J, Liu J, Lu K, Kuo WL, Smith-McCune K, Fishman D, Gray JW *et al*: **Amplification of MDS1/EVI1 and EVI1, located in the 3q26.2 amplicon, is associated with favorable patient prognosis in ovarian cancer.** *Cancer Res* 2007, **67**(7):3074-3084.

**3.** Nanjundan M, Cheng KW, Zhang F, Lahad J, Kuo WL, Schmandt R, Smith-McCune K, Fishman D, Gray JW, Mills GB: **Overexpression of SnoN/SkiL, amplified at the 3q26.2 locus, in ovarian cancers: a role in ovarian pathogenesis.** *Mol Oncol* 2008, **2**(2):164-181.

**4.** Mermel CH, Schumacher SE, Hill B, Meyerson ML, Beroukhim R, Getz G: **GISTIC2.0 facilitates sensitive and confident localization of the targets of focal somatic copy-number alteration in human cancers.** *Genome Biol* 2011, **12**(4):R41.

**5.** Olshen AB, Venkatraman ES, Lucito R, Wigler M: **Circular binary segmentation for the analysis of array-based DNA copy number data.** *Biostatistics* 2004, **5**(4):557-572.
