# Supplementary material for: Phospholipid Scramblase 1, an interferon-regulated gene located at 3q23, is regulated by SnoN/SkiL in ovarian cancer cells
Source: Mol Cancer. 2013 Apr 26;12:32. doi: 10.1186/1476-4598-12-32 (PMC3644492; doi:10.1186/1476-4598-12-32)
Supplement: Additional file 2: Table S1 — DNA Copy Number Variation in PLSCR1 and SnoN across multiple ovarian cancer cell lines. Forty-seven ovarian cancer cell lines were assessed for CNV in PLSCR1 and SnoN. There does not appear to be significant copy number changes for both genes in the cell lines presented where the copy number is nearly invariable from the normal copy number. [file 1476-4598-12-32-S2.pptx]

## Slide 1
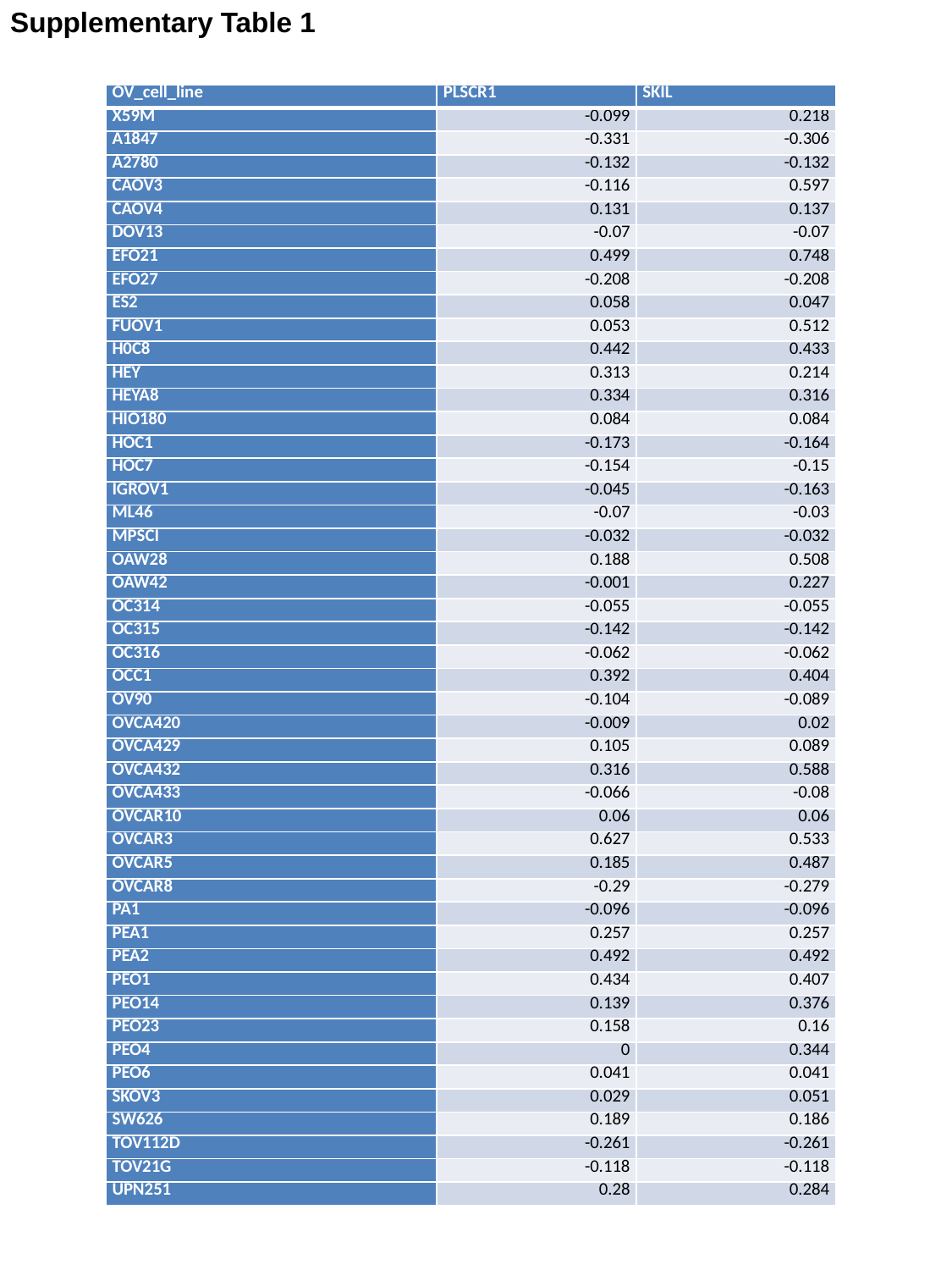

Supplementary Table 1
| OV\_cell\_line | PLSCR1 | SKIL |
| --- | --- | --- |
| X59M | -0.099 | 0.218 |
| A1847 | -0.331 | -0.306 |
| A2780 | -0.132 | -0.132 |
| CAOV3 | -0.116 | 0.597 |
| CAOV4 | 0.131 | 0.137 |
| DOV13 | -0.07 | -0.07 |
| EFO21 | 0.499 | 0.748 |
| EFO27 | -0.208 | -0.208 |
| ES2 | 0.058 | 0.047 |
| FUOV1 | 0.053 | 0.512 |
| H0C8 | 0.442 | 0.433 |
| HEY | 0.313 | 0.214 |
| HEYA8 | 0.334 | 0.316 |
| HIO180 | 0.084 | 0.084 |
| HOC1 | -0.173 | -0.164 |
| HOC7 | -0.154 | -0.15 |
| IGROV1 | -0.045 | -0.163 |
| ML46 | -0.07 | -0.03 |
| MPSCI | -0.032 | -0.032 |
| OAW28 | 0.188 | 0.508 |
| OAW42 | -0.001 | 0.227 |
| OC314 | -0.055 | -0.055 |
| OC315 | -0.142 | -0.142 |
| OC316 | -0.062 | -0.062 |
| OCC1 | 0.392 | 0.404 |
| OV90 | -0.104 | -0.089 |
| OVCA420 | -0.009 | 0.02 |
| OVCA429 | 0.105 | 0.089 |
| OVCA432 | 0.316 | 0.588 |
| OVCA433 | -0.066 | -0.08 |
| OVCAR10 | 0.06 | 0.06 |
| OVCAR3 | 0.627 | 0.533 |
| OVCAR5 | 0.185 | 0.487 |
| OVCAR8 | -0.29 | -0.279 |
| PA1 | -0.096 | -0.096 |
| PEA1 | 0.257 | 0.257 |
| PEA2 | 0.492 | 0.492 |
| PEO1 | 0.434 | 0.407 |
| PEO14 | 0.139 | 0.376 |
| PEO23 | 0.158 | 0.16 |
| PEO4 | 0 | 0.344 |
| PEO6 | 0.041 | 0.041 |
| SKOV3 | 0.029 | 0.051 |
| SW626 | 0.189 | 0.186 |
| TOV112D | -0.261 | -0.261 |
| TOV21G | -0.118 | -0.118 |
| UPN251 | 0.28 | 0.284 |
