# Supplementary material for: Phospholipid Scramblase 1, an interferon-regulated gene located at 3q23, is regulated by SnoN/SkiL in ovarian cancer cells
Source: Mol Cancer. 2013 Apr 26;12:32. doi: 10.1186/1476-4598-12-32 (PMC3644492; doi:10.1186/1476-4598-12-32)
Supplement: Additional file 3: Figure S1 — Induction of PLSCR1 protein expression in response to IFN-2α in a series of resistant and sensitive pancreatic cancer cell lines. (A) IFN-2α treatment of AsPC-1 cells and PANC-1 cells. (B) IFN-2α treatment of BxPC-3 cells and MIA PaCa-2 cells. Cell lysates were harvested from cell lines described in (A) and (B) followed by western analyses for the indicated antibodies. (C) Cell lysates were harvested from IFN-2α treated MIA PaCa-2 cells followed by western analyses for the indicated antibodies. (D) RNA was isolated from IFN-2α treated MIA PaCa-2 cells followed by real-time PCR analyses to quantify PLSCR1 mRNA levels. (E) and (F) Growth assays were performed in AsPC-1, PANC-1, BxPC-3, and MIA PaCa-2 cells in response to IFN-2α treatment. [file 1476-4598-12-32-S3.pptx]

## Slide 1
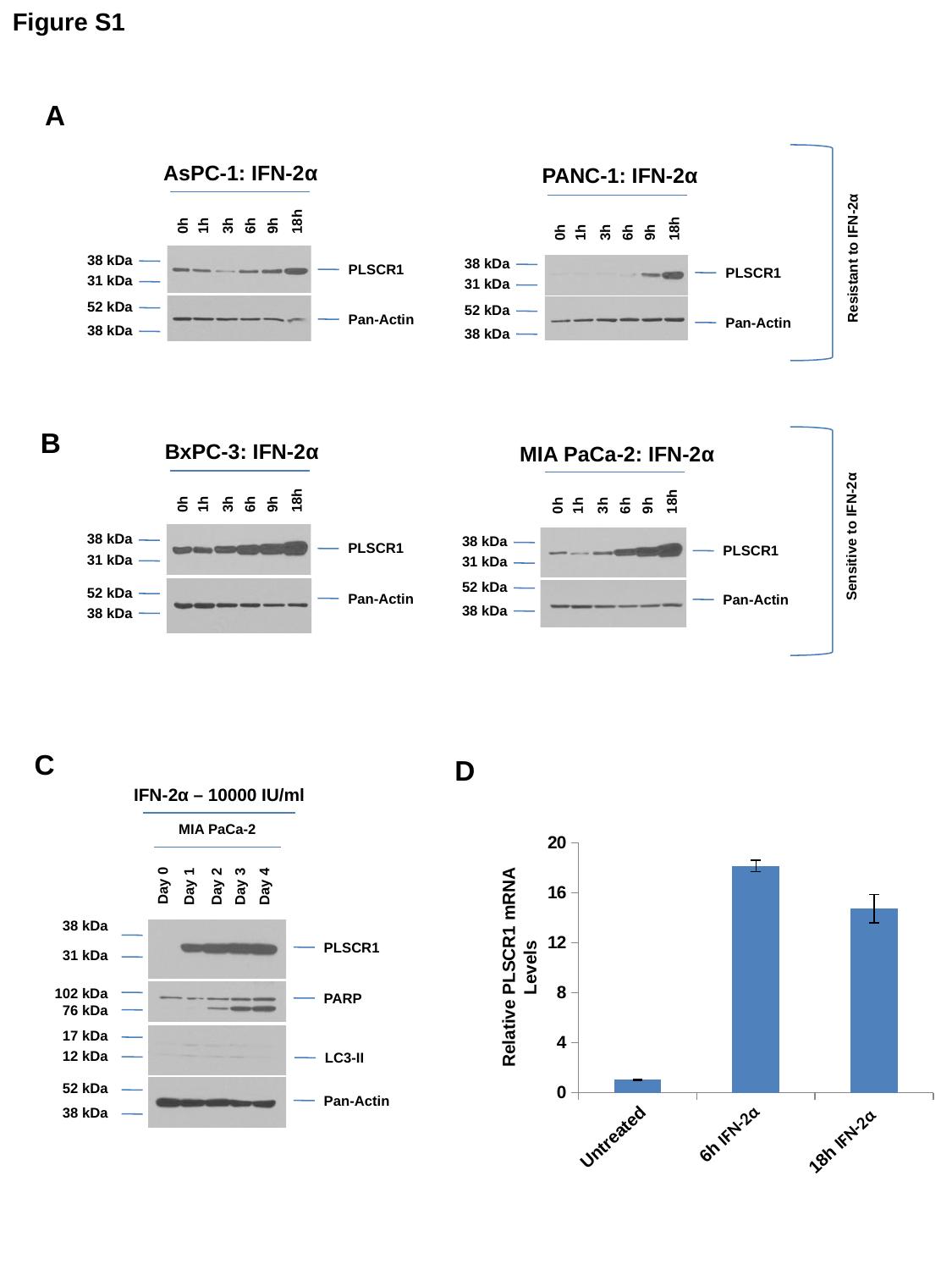

Figure S1
A
PANC-1: IFN-2α
6h
3h
9h
1h
0h
18h
 38 kDa
PLSCR1
 31 kDa
 52 kDa
Pan-Actin
 38 kDa
AsPC-1: IFN-2α
6h
3h
9h
1h
0h
18h
Resistant to IFN-2α
 38 kDa
PLSCR1
 31 kDa
 52 kDa
Pan-Actin
 38 kDa
BxPC-3: IFN-2α
6h
3h
9h
1h
0h
18h
 38 kDa
PLSCR1
 31 kDa
 52 kDa
Pan-Actin
 38 kDa
MIA PaCa-2: IFN-2α
6h
3h
9h
1h
0h
18h
 38 kDa
PLSCR1
 31 kDa
 52 kDa
Pan-Actin
 38 kDa
B
Sensitive to IFN-2α
C
D
IFN-2α – 10000 IU/ml
MIA PaCa-2
Day 3
Day 2
Day 4
Day 1
Day 0
 38 kDa
PLSCR1
 31 kDa
102 kDa
PARP
 76 kDa
 17 kDa
 12 kDa
LC3-II
 52 kDa
Pan-Actin
 38 kDa
### Chart
| Category | |
|---|---|
| Mia None | 1.0402250138487321 |
| Mia 6h IFN | 18.155328354142316 |
| Mia 18h IFN | 14.743292231224451 |Relative PLSCR1 mRNA Levels
6h IFN-2α
Untreated
18h IFN-2α

## Slide 2
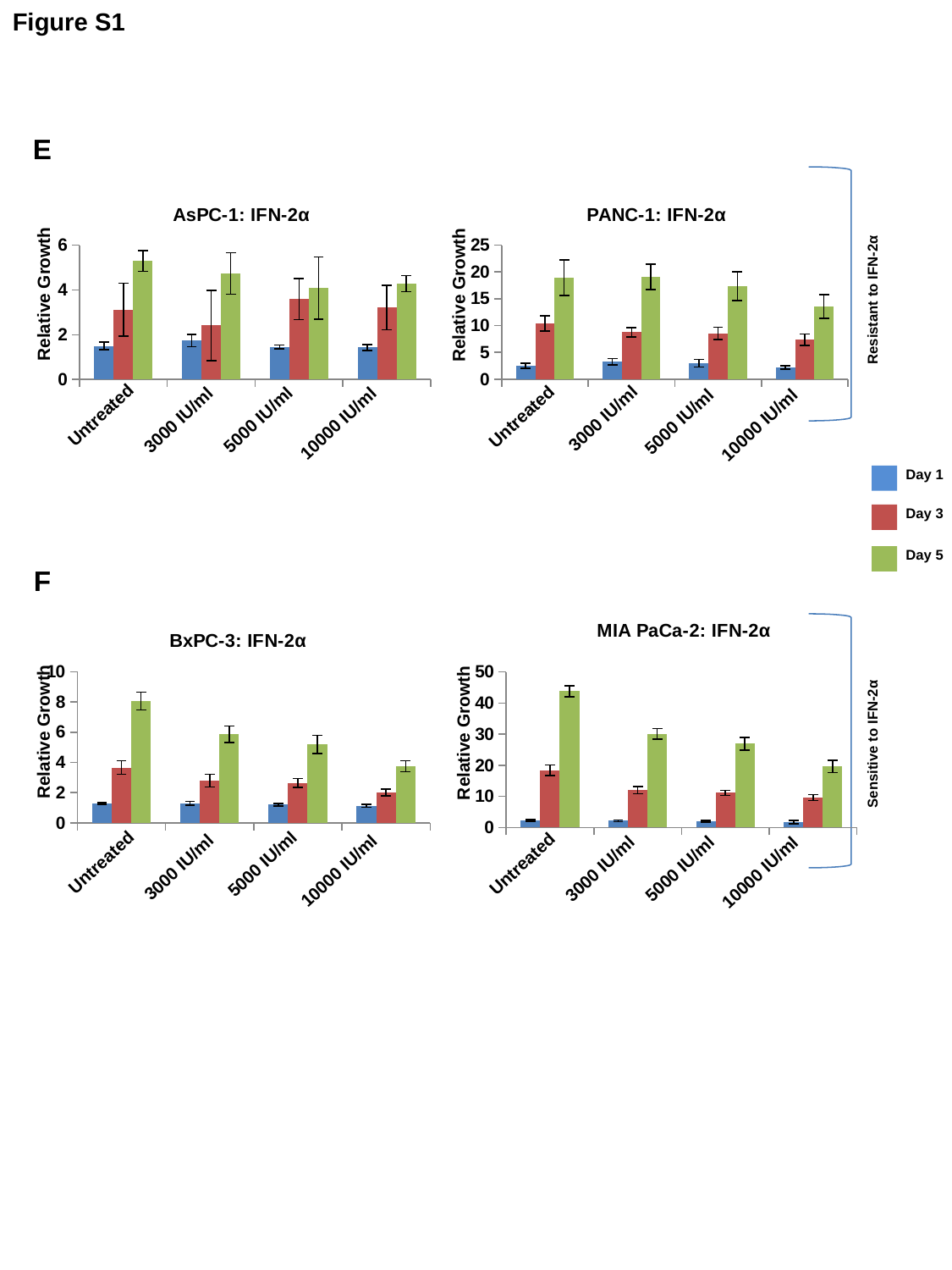

Figure S1
E
### Chart: AsPC-1: IFN-2α
| Category | day 1 | day 3 | day 5 |
|---|---|---|---|
| 0 IFN | 1.499999999999996 | 3.1171052631578946 | 5.292105263157883 |
| 3000 IFN | 1.7350877192982461 | 2.411842105263147 | 4.733333333333348 |
| 5000 IFN | 1.451315789473684 | 3.5938596491228068 | 4.088157894736841 |
| 10000 IFN | 1.429385964912283 | 3.207017543859656 | 4.285087719298246 |
### Chart: PANC-1: IFN-2α
| Category | Day 1 | Day 3 | Day5 |
|---|---|---|---|
| None | 2.498599439775911 | 10.400560224089665 | 18.944677871148425 |
| 3000 IU | 3.2023809523809605 | 8.744747899159664 | 19.100840336134453 |
| 5000IU | 2.9562324929971977 | 8.516456582633054 | 17.353641456582633 |
| 10000IU | 2.150910364145658 | 7.348739495798333 | 13.565826330532232 |Relative Growth
Relative Growth
Resistant to IFN-2α
Untreated
Untreated
3000 IU/ml
5000 IU/ml
3000 IU/ml
5000 IU/ml
10000 IU/ml
10000 IU/ml
Day 1
Day 3
Day 5
F
### Chart: BxPC-3: IFN-2α
| Category | day 1 | day 3 | day 5 |
|---|---|---|---|
| 0 IFN | 1.2792008196721312 | 3.654030054644809 | 8.058401639344282 |
| 3000 IFN | 1.2966188524590159 | 2.7950819672131146 | 5.8490437158469994 |
| 5000 IFN | 1.1992827868852487 | 2.647882513661202 | 5.176229508196721 |
| 10000 IFN | 1.137465846994536 | 2.0143442622950842 | 3.7428278688524683 |
### Chart: MIA PaCa-2: IFN-2α
| Category | Day 1 | Day 3 | Day5 |
|---|---|---|---|
| None | 2.263182674199624 | 18.410075329566865 | 43.805084745762564 |
| 3000 IU | 2.171845574387947 | 12.018361581920878 | 30.11770244821093 |
| 5000IU | 2.060263653483993 | 11.13653483992471 | 26.91996233521662 |
| 10000IU | 1.7645951035781546 | 9.678436911487783 | 19.6195856873823 |
Relative Growth
Relative Growth
Sensitive to IFN-2α
Untreated
Untreated
3000 IU/ml
5000 IU/ml
3000 IU/ml
5000 IU/ml
10000 IU/ml
10000 IU/ml
