# Supplementary material for: Phospholipid Scramblase 1, an interferon-regulated gene located at 3q23, is regulated by SnoN/SkiL in ovarian cancer cells
Source: Mol Cancer. 2013 Apr 26;12:32. doi: 10.1186/1476-4598-12-32 (PMC3644492; doi:10.1186/1476-4598-12-32)
Supplement: Additional file 4: Figure S2 — No effect on PLSCR1 protein levels upon NAC treatment or SnoN knockdown in the absence/presence of IFN-2α. (A) HEY cells were treated with 3000 IU/ml IFN-2α (0, 1, 3 hours). RNA was isolated followed by real-time PCR analysis to quantify SnoN mRNA levels. (B) HEY cells were treated with IFN-2α, NAC, or IFN-2α in combination with NAC at the specified doses. Cell lysates were harvested and analyzed by western blotting analyses for the indicated antibodies. (C) HEY cells were transfected with SnoN siRNA and treated with/without IFN-2α. Cell lysates were harvested and western analyses performed for the indicated antibodies. [file 1476-4598-12-32-S4.pptx]

## Slide 1
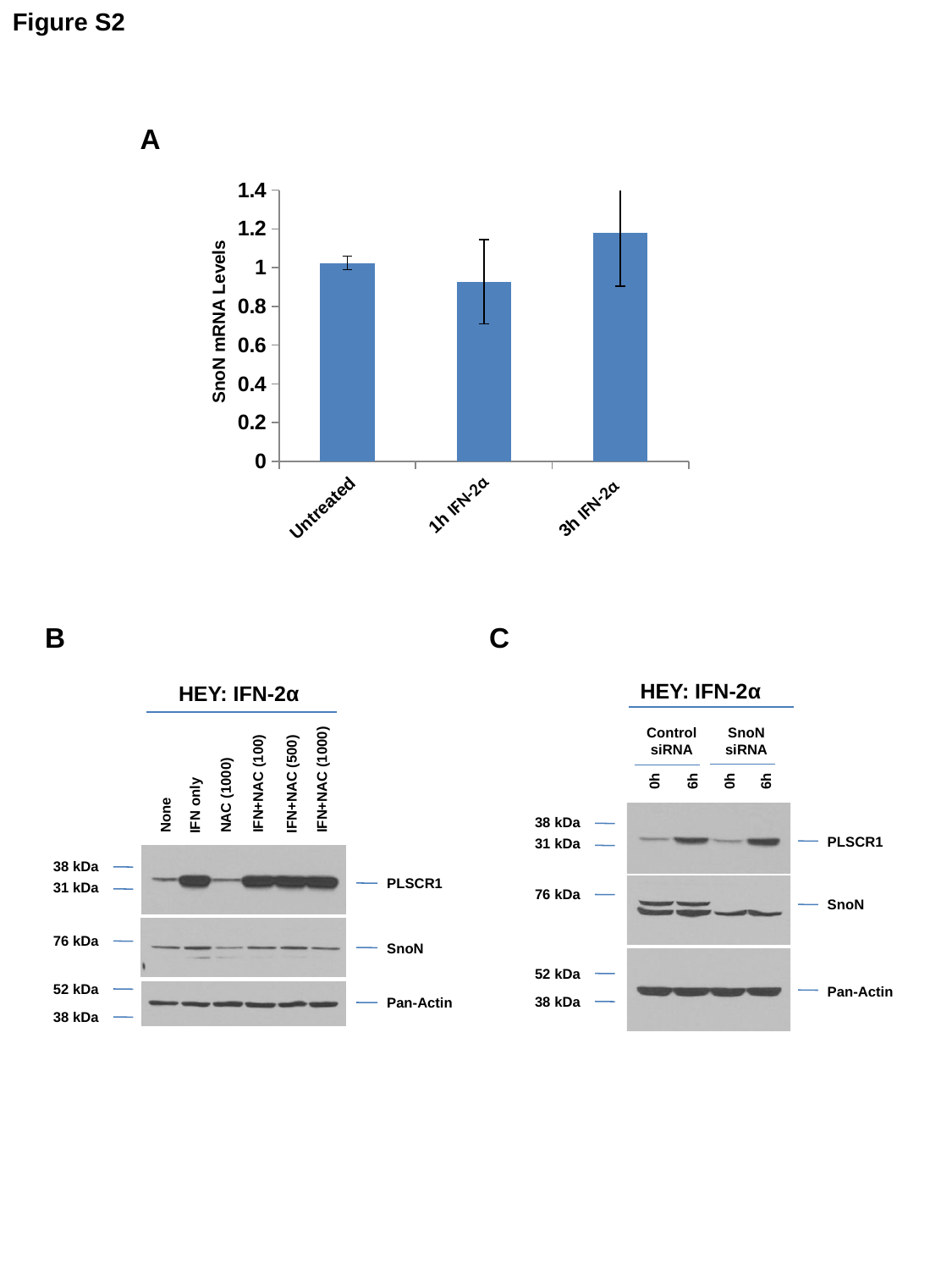

Figure S2
A
### Chart
| Category | |
|---|---|
| Hey None | 1.02506991328868 |
| Hey 1h IFN | 0.9276693691321262 |
| Hey 3h IFN | 1.1812313980461553 |SnoN mRNA Levels
1h IFN-2α
Untreated
3h IFN-2α
C
B
HEY: IFN-2α
HEY: IFN-2α
Control
siRNA
SnoN
siRNA
6h
6h
IFN+NAC (1000)
0h
0h
IFN+NAC (100)
IFN+NAC (500)
NAC (1000)
IFN only
None
 38 kDa
PLSCR1
 31 kDa
 38 kDa
PLSCR1
 31 kDa
 76 kDa
SnoN
 76 kDa
SnoN
 52 kDa
 52 kDa
Pan-Actin
 38 kDa
Pan-Actin
 38 kDa
